# Correction: Systematic synthesis of bisected *N*-glycans and unique recognitions by glycan-binding proteins

**DOI:** 10.1039/d2sc90223k

**Published:** 2022-11-09

**Authors:** Xuefeng Cao, Shuaishuai Wang, Madhusudhan Reddy Gadi, Ding Liu, Peng G. Wang, Xiu-Feng Wan, Jian Zhang, Xi Chen, Lauren E. Pepi, Parastoo Azadi, Lei Li

**Affiliations:** Department of Chemistry, Georgia State University Atlanta GA USA lli22@gsu.edu; MU Center for Research on Influenza Systems Biology (CRISB), University of Missouri Columbia MO USA; Department of Molecular Microbiology and Immunology, School of Medicine, University of Missouri Columbia MO USA; Bond Life Sciences Center, University of Missouri Columbia MO USA; Department of Electrical Engineering & Computer Science, College of Engineering, University of Missouri Columbia MO USA; Z Biotech, LLC Aurora CO USA; Department of Chemistry, University of California One Shields Avenue Davis CA USA; Complex Carbohydrate Research Center, University of Georgia Athens GA USA

## Abstract

Correction for ‘Systematic synthesis of bisected *N*-glycans and unique recognitions by glycan-binding proteins’ by Xuefeng Cao *et al.*, *Chem. Sci.*, 2022, **13**, 7644–7656, https://doi.org/10.1039/D1SC05435J.

The authors regret that funding details were incorrect in the acknowledgements section of the original article. The corrected acknowledgements section for this article is shown below.

**Acknowledgements**

The work was supported by United States National Institutes of Health (NIH) awards (U54HL142019 to LL, R44GM123820 to LL and JZ, and R21AI144433 to XFW). The work is partially supported by GlycoMIP, a National Science Foundation Materials Innovation Platform funded through Cooperative Agreement DMR-1933525 and NIH R24GM137782. The glycan microarray analysis software MotifFinder was developed under the support of NIH SBIR award R43GM131430 to JZ.

The Royal Society of Chemistry apologises for these errors and any consequent inconvenience to authors and readers.

## Supplementary Material

